# Improving TFT Device Performance by Changing the Thickness of the LZTO/ZTO Dual Active Layer

**DOI:** 10.3390/mi15101235

**Published:** 2024-09-30

**Authors:** Liang Guo, Suhao Wang, Xuefeng Chu, Chao Wang, Yaodan Chi, Xiaotian Yang

**Affiliations:** 1 Key Laboratory of Architectural Cold Climate Energy Management, Ministry of Education, Jilin Jianzhu University, Changchun 130118, China; guoliang@jlju.edu.cn (L.G.); wangsuhao5038@163.com (S.W.); stone2009@126.com (X.C.); chiyaodan@jlju.edu.cn (Y.C.); 2 Department of Basic Science, Jilin Jianzhu University, Changchun 130118, China; 3 School of Electrical and Computer Science, Jilin Jianzhu University, Changchun 130118, China; 4 Department of Chemistry, Jilin Normal University, Siping 136000, China

**Keywords:** dual layer LZTO/ZTO TFT, XPS analysis, changing thickness

## Abstract

The primary objective of this research paper is to explore strategies for enhancing the electrical performance of dual active layer thin film transistors (TFTs) utilizing LZTO/ZTO as the bilayer architecture. By systematically adjusting the thickness of the active layers, we achieved significant improvements in the performance of the LZTO/ZTO TFTs. An XPS analysis was performed to elucidate the impact of the varying O_2_ element distribution ratio within the LZTO/ZTO bilayer thin film on the TFTs performance, which was directly influenced by the modification in the active layer thickness. Furthermore, we utilized atomic force microscopy to analyze the effect of altering the active layer thickness on the surface roughness of the LZTO/ZTO bilayer film and the impact of this roughness on the TFTs electrical performance. Through the optimization of the ZTO active layer thickness, the LZTO/ZTO TFT exhibited an mobility of 10.26 cm^2^ V^−1^ s^−1^ and a switching current ratio of 5.7 × 10^7^, thus highlighting the effectiveness of our approach in enhancing the electrical characteristics of the TFT device.

## 1. Introduction

Amorphous metal oxide (AOS) thin-film transistors (TFTs) have garnered significant attention in the realm of display technology owing to their exceptional characteristics, including high carrier mobility, substantial open-state current, minimal off-state current, and commendable transmittance in the visible spectrum [1,2,3]. Amorphous metal oxide IGZO TFTs have been extensively investigated and fabricated for applications in display technology. However, the cost of In and Ga, along with their toxic properties, cannot be overlooked, necessitating the search for alternative elements to replace In and Ga [4]. Given that the ionic valence of Sn closely resembles that of In, and its abundance in the Earth’s crust renders Sn ions a viable alternative to In ions, amorphous metal oxide ZnSnO TFTs are currently garnering significant interest due to their favorable optical and electrical properties [5]. However, ZTO TFTs, despite their high mobility, exhibit a negative threshold voltage, thereby restricting their broad applicability [6]. Therefore, how to improve the performance of ZTO TFTs to meet the standards for application in the display field is still a challenge [7].

Doping of intrinsic TFTs with metallic elements and preparation of dual active layer TFTs are considered the best options to improve the performance of amorphous metal oxides [8]. In recent years, strategies for channel design, active layer thickness, and annealing temperature have been reported for single-layer oxides. The appropriate active layer thickness is an important factor that affects the saturation mobility as well as the threshold voltage stability of TFT devices [9,10,11]. The change in the active layer’s thickness affects the movement of free electrons in the film as well as the percentage of oxygen vacancies. Lower oxygen vacancies not only increase the saturation migration of TFT devices but also reduce the threshold voltage of TFT devices to enhance the stability of the devices [12,13]. The change in the thickness of the active layer changes the surface structure of the film to some extent, affecting the roughness of the film surface. This affects the interfacial effects of the film, causing the number and rate of movement of the free electrons within the film to change. This changes the electrical properties of the TFT [10,14]. Since Zn leads to higher oxygen vacancy formation, Li is doped on the basis of ZTO TFTs. Li ions replace Zn ions to form Li-O bonds to reduce oxygen vacancies to improve the stability of ZTO TFTs. This improves the negative threshold voltage problem of ZTO TFTs because of the involvement of Li ions. However, the disadvantage of the substantial decrease in the mobility of ZTO TFTs during the Li ion participation cannot be neglected. Therefore, LZTO/ZTO dual-active-layer TFTs were prepared on the basis of two single-layer TFTs. Electrical performance is improved by combining the advantages of the semiconductor front and rear channels. This satisfies both the high mobility and the stable threshold voltage [15,16].

In this study, the thickness of the zinc tin oxide (ZTO) film within a dual-active-layer structure was systematically varied using magnetron sputtering under identical preparation conditions. LZTO/ZTO dual-active-layer thin-film transistors (TFTs) with varying active layer thicknesses were fabricated. The impact of these thickness alterations on key transistor parameters, including carrier mobility (μ), switching current ratio, threshold voltage (Vth), and subthreshold swing (SS), was comprehensively investigated. Additionally, X-ray photoelectron spectroscopy (XPS) analysis was conducted to evaluate the oxygen vacancy (VO) concentrations in films of different thicknesses. Atomic force microscopy (AFM) measurements were also performed to assess the surface roughness of the films. The findings indicate that alterations in the active layer’s thickness result in changes in the oxygen vacancy concentrations and surface roughness of the films, which significantly influence the overall performance of the TFTs.

## 2. Experimental Process

In this paper, magnetron sputtering is used in the experiments to prepare dual active layer TFTs. The TFTs were prepared using 285 nm thick SiO_2_/p-Si as a substrate. The SiO_2_/p-Si substrate was cleaned under ultrasound for 10 min according to acetone, anhydrous ethanol, and deionised water sequentially. After cleaning and blow-drying with nitrogen, the reverse gel lithography process is carried out on the cleaned SiO_2_/p-Si substrate. All samples were prepared with TFTs with an electrode width of 300 μm and a channel spacing of 10 μm on SiO_2_/P-Si with a thickness of 285 nm. A dual-active-layer LZTO/ZTO TFT was prepared by the magnetron sputtering method. The lower active layer was prepared by using a ZnSnO (70%:30%) target sputtered at 85 W power for 2, 3, 4 and 5 min respectively. The upper active layer was prepared by sputtering at 85 W power for 10 min using ZnSnO:Li (70%:30%) target. The different ZTO sputtering times represent samples I, II, III, and IV, respectively. The active layer thicknesses of the four samples were 48 nm, 52 nm, 56 nm, and 60 nm, in that order. A vacuum pressure of 9.8 × 10^−4^ (Pa) is maintained in the cavity before magnetron sputtering. The ratio of Ar_2_ to O_2_ during sputtering was 90%:10%. After sputtering, the TFTs anneal at 600 °C for 1 h under air conditions. Al electrodes with a thickness of 50 nm were evaporated on the LZTO/ZTO active layer as source and drain electrodes by electron beam evaporation.

## 3. Results and Discussion

**Figure 1:**
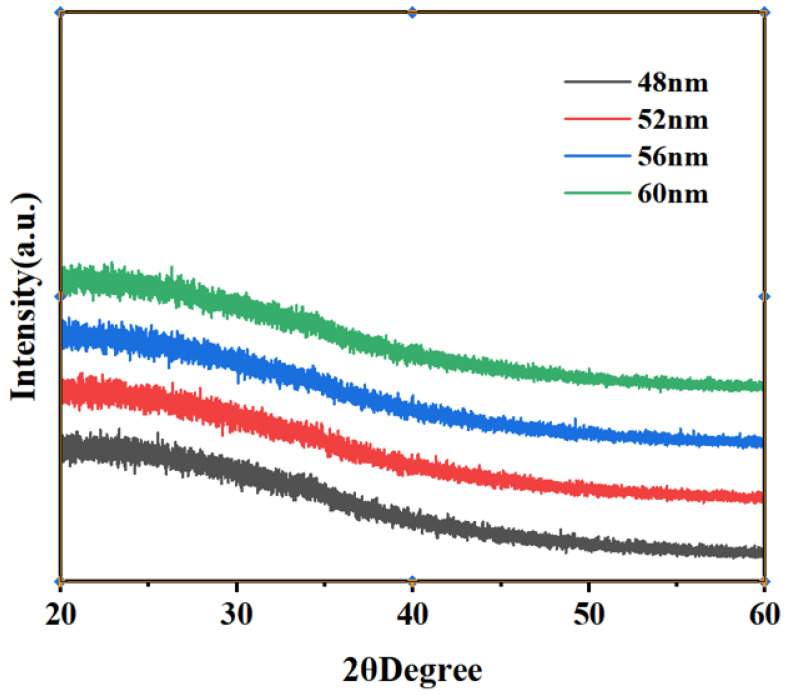
XRD analysis of thicknesses of 48, 52, 56, and 60 nm.

Figure 1 shows the XRD test results of different samples. XRD analysis of the four samples was able to obtain the effect of varying the thickness of the active layer on the internal structure of the film. The absence of diffraction peaks for all four samples can be seen in Figure 1. This indicates that the films of LZTO/ZTO at different thicknesses have an amorphous structure. It is also shown that the change in the thickness of the active layer does not affect the diffraction peaks of the LZTO/ZTO films. This indicates that the film is uniform across the different active layer thicknesses [17,18].

**Figure 2:**
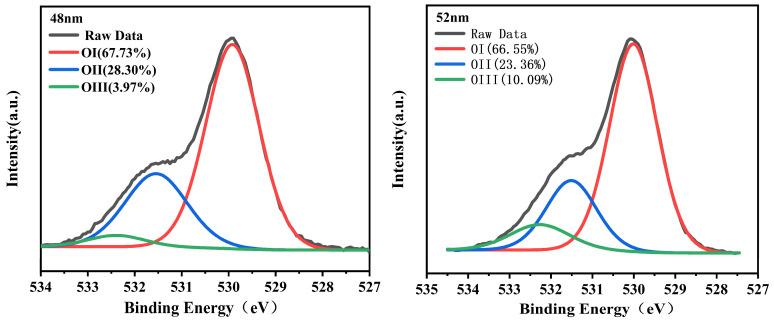

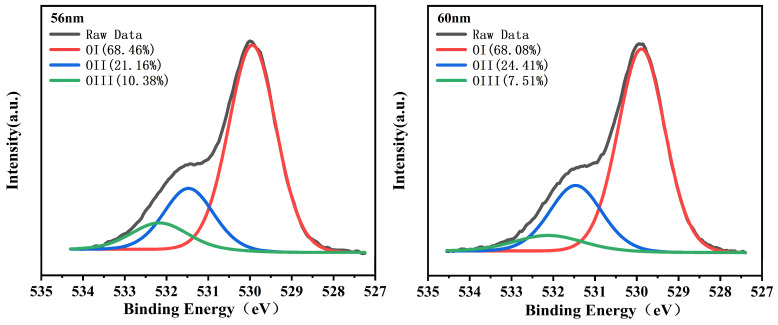
XPS analysis of thicknesses of 48, 52, 56, and 60 nm. OI represents the O bond in the metal, OII represents the oxygen vacancy, and OIII represents the oxygen in the hydroxyl group.

Changes in the thickness of the ZTO film affect the defects and carrier concentration in the active layer. The saturation mobility changes as the thickness of the ZTO film changes. The percentage of different forms of oxygen in the active layer changes as the thickness of the active layer changes. The oxygen vacancies have a strong influence on the carrier concentration and propagation rate. When the oxygen vacancies are too large, oxygen ions will escape from the lattice and impede the charge propagation in the active layer, reducing the carrier transfer rate and thus the carrier mobility [19]. In order to understand the percentage of different forms of oxygen in the film as the thickness of the ZTO film changes, X-ray electron spectroscopy (XPS) was used to further analyze the change in the proportion of different forms of oxygen. The oxygen percentage of different samples is shown in Figure 2.

The various oxygen species within the film were segregated into three distinct sub-peaks through Gaussian fitting. Specifically, OI represents the peak position of the metal-oxygen bond at 529.94 ± 0.07 eV, OII corresponds to the oxygen vacancy peak positioned at 531.51 ± 0.05 eV, and OIII denotes the oxygen-hydrogen bond peak at 532.27 ± 0.16 eV [20,21,22]. Figure 2 reveals a discernible trend wherein the oxygen vacancy concentration reduces from 28.30% to 21.16% as the thickness of the active layer increases, prior to sample III. This decrement in oxygen vacancies corresponds to a decrease in film defects, which weakens the active layer’s adsorption effect on free electrons, enhancing the carrier concentration and ultimately bolstering the LZTO/ZTO TFT performance. However, following 56 nm, as the active layer thickness further increases, the oxygen vacancy concentration rises from 21.16% to 24.41%. This rise in film defects leads to an increased number of oxygen ions being separated from the metal lattice, adsorbing electrons within the film, thereby reducing the carrier concentration and diminishing the TFT device’s performance [23]. XPS analysis concludes that the oxygen content proportion in the film is closely linked to the thickness of the active layer film [24]. Consequently, identifying the optimal active layer thickness emerges as a crucial factor in enhancing the performance of TFT devices.

**Figure 3:**
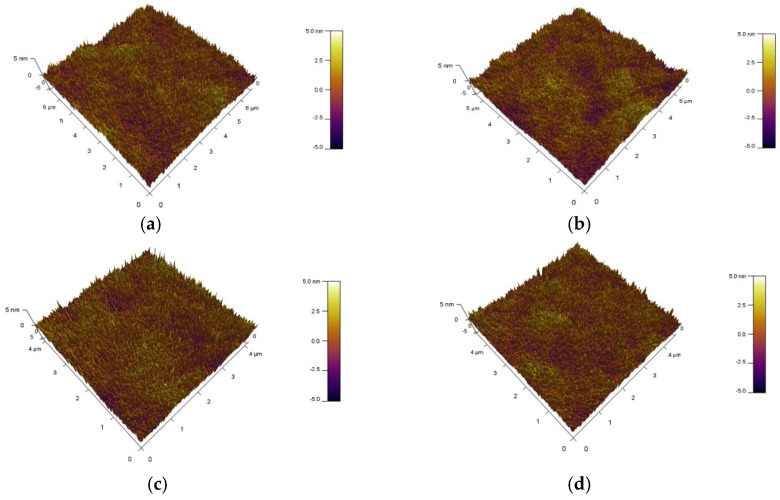
AFM characterization scan. Images of thin films with different thicknesses (**a**) 48 nm, (**b**) 52 nm, (**c**) 56 nm, and (**d**) 60 nm.

In order to investigate the effect of different active layer film thicknesses on the morphology and roughness of the film surface, atomic force microscopy (AFM) tests were performed on each of the four samples. The following Figure 3 shows the surface morphology of different samples. Table 1 shows the roughness (RSM) of different samples. After testing, it was found that by changing the film thickness of the active layer, the surface roughness of the film is greatly affected [25]. The roughness of the film affects the interfacial effects of the film. The greater the roughness of the film the more pronounced the interfacial effect of the film. Interfacial effects in thin films can have an impact on carrier scattering and carrier trapping in thin films. The interfacial effect of the film is reduced when the film roughness is small, resulting in reduced carrier scattering and trapping effects in the film and enhanced electrical performance of the TFT device [14]. From Table 1, it can be seen that the lowest film roughness of 0.74 nm was found in the four samples with a thickness of 56 nm. Before the thickness of the TFT reaches 56 nm, the film roughness decreases as the thickness increases. After the thickness of the TFT reaches 56 nm, the film roughness increases as the thickness further increases. This also shows that the appropriate active layer thickness will have an effect on the roughness of the film surface [26,27,28].

**Figure 4:**
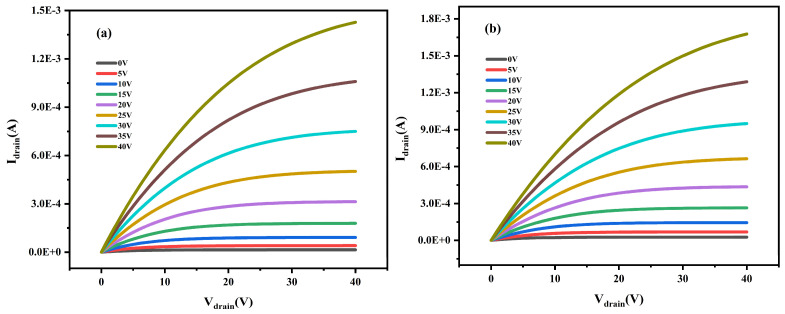

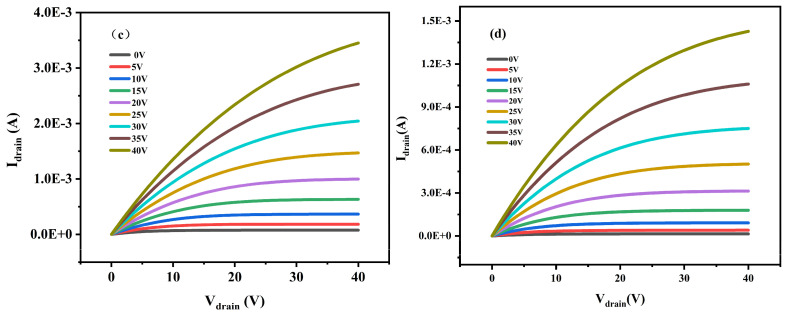
(**a**–**d**) The output characteristic curves of four samples respectively.

In order to analyze the electrical performance parameters of different samples, four samples were tested for electrical performance using a semiconductor parameter analyzer (B1500A, Santa Rosa, CA, USA). Figure 4 presents the output characteristic curves of four samples. As you can see from the graph, I_DS_ is increasing as V_DS_ increases, and as V_DS_ gradually increases, I_DS_ tends to be saturated. This indicates that LZTO/ZTO thin-film transistors are n-type semiconductor devices.

**Figure 5:**
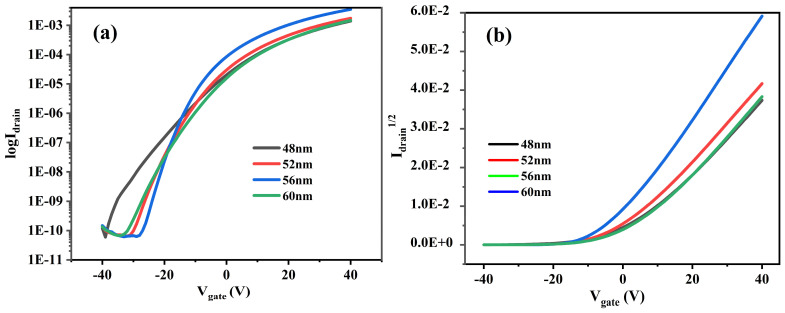
(**a**,**b**) Transfer characteristic curves of TFTs with different thicknesses.

Figure 5 shows the transfer characteristic curves of V_GS_ for four samples in the range of −40 V to 40 V. It can be seen from the figure that the transfer characteristic curve shifts when the thickness of the active layer is varied. The results show that the performance of TFTs can be greatly affected by changing the active layer thickness of ZTO/LZTO [29]. The LZTO/ZTO TFT’s electrical performance parameters extracted from Figure 5 are presented in Table 2.

The threshold voltage (V_TH_) of the TFT device can be obtained by fitting the linear part of the transfer characteristic curve. The saturation mobility of the device can be obtained by fitting Equation (1), where Ci is the gate capacitance per unit area, W is a constant indicating the channel width, *L* is a constant indicating the channel length, and *V_GS_* and *I_DS_* are denoted as the gate voltage and drain current, respectively.

(1) $$\mu_{SAT}=\frac{2L}{WC_{i}}{\left(\frac{\partial \sqrt{I_{DS}}}{\partial V_{GS}}\right)}^{2}$$

(2) $$I_{ON}/I_{OFF}=\frac{{\left(I_{DS}\right)}_{\max}}{{\left(I_{DS}\right)}_{\min}}$$

(3) $$SS=\frac{dV_{GS}}{d(\log I_{DS})}$$

The switching current ratio of the TFT device can be fitted by Equation (2) and the subthreshold swing fitting results of the TFT device can be fitted by Equation (3) are presented in Table 2.

From Table 2, it can be seen that the mobility of the four samples firstly increases and then decreases with the increase in the thickness of the active layer. The mobility of the TFT with a thickness of 56 nm can reach up to 10.26 cm^2^ V^−1^ S^−1^, and the trend of this mobility is related to the defects within the film. The oxygen vacancies within the film decrease with the increasing thickness of the active layer before the film thickness increases from 48 nm to 56 nm. The reduction in defects inside the film is less of an obstacle to the movement of carriers in the active layer and therefore the mobility increases. When the film thickness increases from 56 nm to 60 nm, the oxygen vacancies within the film increase again. This is due to the increase in defects within the film oxygen atoms escape from the lattice adsorbing free electrons from the active layer. This limits the movement of free electrons in the active layer leading to a decrease in device saturation mobility. In addition to this, the roughness of the film surface changes as the thickness of the active layer changes. The surface roughness of the four samples in Table 1 decreases and then increases, and the film with a thickness of 56 nm has the lowest surface roughness of 0.74 nm. The weakening of the interfacial effect of the film at a lower roughness reduces the trapping of free electrons in the active layer by the film, thus leading to an increase in the mobility. It is verified that different roughnesses cause the electrical properties of TFT devices to be altered. Changes in the thickness of the active layer can also affect the backplane conductivity of the TFT, leading to a reduction in the off-state current, affecting the switching current ratio of the TFT. The right thickness of the active layer enables the TFT to obtain the best electrical performance, which is also in line with the variation of the switching current ratio of the TFT in Table 2.

By analyzing the effect of changing the thickness of the LZTO/ZTO active layer on the electrical properties, it can be found the thickness has a significant effect on the electrical properties of TFTs [30]. This is because the change in the thickness of the active layer can affect the structure of the film. As a result, the movement of the free electrons in the active layer changes [31]. When the active layer film is too thin, the voids and defects in the film increase to adsorb the free electrons in the film. This hinders the movement of free electrons and is not conducive to the conduction of the current in the film. When the active layer film is too thick, it affects the degree of carrier depletion in the film. As a result, current pinch-off and saturation characteristics cannot be generated, resulting in the poor electrical properties of the film. Thus, a suitable active layer thickness can deplete free electrons without affecting current conduction in the film.

## 4. Conclusions

LZTO/ZTO dual-active-layer TFTs were prepared by magnetron sputtering, successfully combining the advantages of single-layer LZTO and ZTO TFTs. Additionally, four samples were prepared by varying the thickness of the lower ZTO film. XRD, XPS and AFM tests were performed on four dual-active-layer sample films respectively. When the thickness of the active layer is varied, the mobility of the TFT device first increases and then decreases with increasing thickness. The TFT device exhibits the best electrical performance when the film thickness is 56 nm, with μ_SAT_ of 10.26 cm^2^ V^−1^ S^−1^, V_TH_ of −3.49 V, SS of 2.69 and I_ON_/I_OFF_ of 5.7 × 10^7^. The results show that the appropriate active layer thickness has a great influence on the performance oil of dual active layer TFTs. Therefore, it is very important to find the right active layer thickness.

## Figures and Tables

**Table 1:** Film roughness under different samples.

Film Thickness	Surface Roughness/nm
48 nm	0.80
52 nm	0.77
56 nm	0.74
60 nm	0.87

**Table 2:** Electrical performance parameters of different.

Film Thickness	μ_SAT_ (cm^2^/VS)	V_TH_ (V)	SS (V/decade)	I_on_/I_off_
48 nm	5.59	2.92	3.25	2.4 × 10^7^
52 nm	5.77	−0.71	3.42	2.7 × 10^7^
56 nm	10.26	−3.49	2.69	5.7 × 10^7^
60 nm	6.17	3.84	4.28	2.2 × 10^7^

## Data Availability

The data that support the findings of this study are available from the corresponding authors upon reasonable request.
